# Pairwise detection of site-specific receptor phosphorylations using single-molecule blotting

**DOI:** 10.1038/ncomms11107

**Published:** 2016-03-24

**Authors:** Kyung Lock Kim, Daehyung Kim, Seongsil Lee, Su-Jeong Kim, Jung Eun Noh, Joung-Hun Kim, Young Chan Chae, Jong-Bong Lee, Sung Ho Ryu

**Affiliations:** 1 Department of Life Sciences, Pohang University of Science and Technology (POSTECH), Pohang 790-784, Republic of Korea; 2 Department of Physics, Pohang University of Science and Technology (POSTECH), Pohang 790-784, Republic of Korea; 3 School of Interdisciplinary Bioscience and Bioengineering, Pohang University of Science and Technology (POSTECH), Pohang 790-784, Republic of Korea

## Abstract

Post-translational modifications (PTMs) of receptor tyrosine kinases (RTKs) at the plasma membrane (PM) determine the signal transduction efficacy alone and in combination. However, current approaches to identify PTMs provide ensemble results, inherently overlooking combinatorial PTMs in a single polypeptide molecule. Here, we describe a single-molecule blotting (SiMBlot) assay that combines biotinylation of cell surface receptors with single-molecule fluorescence microscopy. This method enables quantitative measurement of the phosphorylation status of individual membrane receptor molecules and colocalization analysis of multiple immunofluorescence signals to directly visualize pairwise site-specific phosphorylation patterns at the single-molecule level. Strikingly, application of SiMBlot to study ligand-dependent epidermal growth factor receptor (EGFR) phosphorylation, which is widely thought to be multi-phosphorylated, reveals that EGFR on cell membranes is hardly multi-phosphorylated, unlike *in vitro* autophosphorylated EGFR. Therefore, we expect SiMBlot to aid understanding of vast combinatorial PTM patterns, which are concealed in ensemble methods, and to broaden knowledge of RTK signaling.

## Abstract (web summary)

Current methods to measure post-translational modifications of receptor tyrosine kinases provide ensemble results. Here the authors develop a single-molecule blotting (SiMBlot) assay that detects site-specific phosphorylation patterns at the single-molecule level.

## Graphical abstract

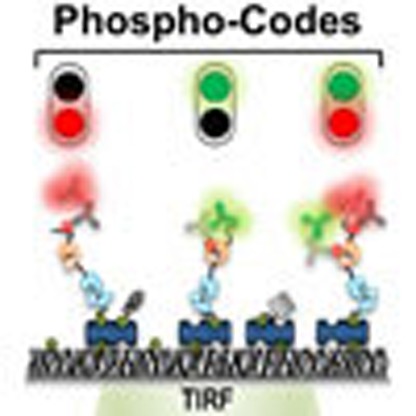

Receptor tyrosine kinases (RTKs) play important roles in diverse biological functions, including cell-to-cell communication, proliferation and signal propagation, by their inherent kinase activity. Most biological processes, including RTK signaling, are coordinated by protein regulation such as post-translational modifications (PTMs), many of which provide binding sites for specific protein–protein interactions and signaling complex formation^1^,^2^. Understanding how signaling receptor molecules are dynamically modified has helped to elucidate their roles in cellular function and regulation^3^,^4^. To determine the characteristics of specific protein pools, conventional methods, such as western blotting and mass spectrometry (MS), are widely used. Tremendous technological advances in biochemical and proteomic approaches achieved the identifications of more than 400 discrete types of modifications and 90,000 individual PTMs^5^. However, existing ensemble methods are virtually inapplicable to detect the combination of PTM sites on a single polypeptide molecule^4^,^6^, the so-called ‘PTM code'^7^, which may confer different properties and functions^8^,^9^,^10^. They suffer from inherent problems including ensemble averaging, loss of intact protein information, stochastic site assignment of combinatorial modification pattern and laborious and high-cost assay. Therefore, analysis of site-specific PTM patterns within individual protein molecules is still unexplored and remains challenging.

Recently, the emerging development of single-molecule techniques enables the observation and characterization of individual molecules for exquisite qualitative and quantitative analysis, avoiding ensemble error^11^,^12^,^13^,^14^. Single-molecule techniques are well suited for characterizing multiple PTMs dispersed along the entire protein sequence^13^,^14^ but no feasible method exists. One promising approach is single-molecule imaging combined with immunofluorescence labeling, which may yield quantitative measurement of PTM status at the single-molecule level. Methods based on super-resolution imaging in intact cells^15^,^16^ cannot control the intrinsic density of interesting protein, preventing the discrimination of individual modified proteins by high molecular density on the PM^17^. Methods based on single-molecule isolation^11^,^12^ can properly control the density of the protein immobilized on the single-molecule surface. However, this seemingly straightforward strategy comes with several practical impediments. First, antibody host species, especially immobilization antibody species, is cumbersome on the selection of antibody sets for multiple immunolabeling. Second, interacting proteins may mask the PTM sites, serving as docking sites for diverse signaling proteins. Third, multiple immunofluorescence labeling on a single polypeptide chain can be prevented by steric hindrance, also known as epitope occlusion. These limitations have hampered the application of single-molecule isolation techniques to the study of combinatorial PTMs.

Here, we have described a simple, ultra-rapid and low-cost single-molecule assay with an antibody-free immobilization to investigate combinatorial PTMs of RTKs, named as ‘Single-Molecule Blotting' (SiMBlot). SiMBlot can directly immobilize biotinylated cell surface proteins on the single-molecule surface and enables the pairwise immunofluorescence labeling to detect multi-site PTMs of a single polypeptide molecule. To demonstrate the unique power of this approach, we apply SiMBlot to reveal the pairwise site-specific phosphorylation patterns of individual EGFR molecules, which are extracted from the cell surface membrane in response to the EGF stimulus or sampled from an *in vitro* autophosphorylation assay. Our results call into question ligand-dependent multi-phosphorylation of EGFR, which is popularly believed to occur^1^,^2^,^18^, and provide an insight into the molecular mechanism underlying EGFR activation.

## Results

### Cell surface protein isolation for single-molecule study

**Figure 1:**
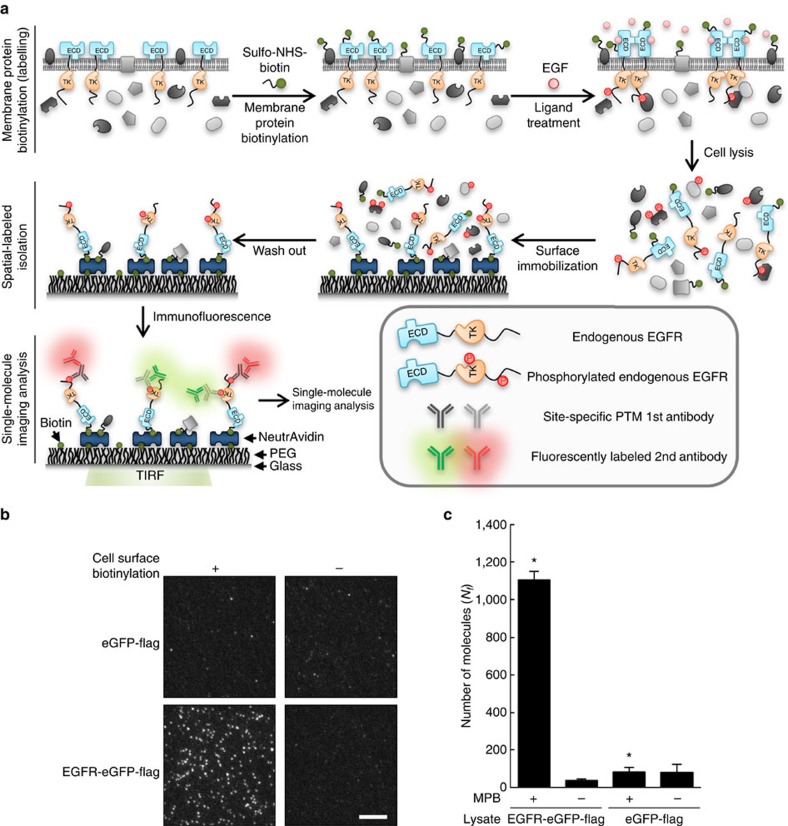
Schematic outline of the SiMBlot assay. (**a**) (top) Cell surface proteins were labeled with a biotin derivative reagent (Sulfo-NHS-Biotin). After ligand treatment, cells were lysed with lysis buffer. (middle) From the cell lysate, biotinylated proteins were tethered to NeutrAvidin on a single-molecule surface. (bottom) Modifications of pulled-down proteins were visualized using total internal reflection fluorescence (TIRF) microscopy, site-specific anti-PTM antibodies, and fluorophore-labeled secondary antibodies. ECD, extracellular domain. TK, tyrosine kinase. (**b**) COS7 cells expressing EGFR-eGFP-flag were labeled with a biotin derivative reagent (Sulfo-NHS-Biotin). Cells were extracted using lysis buffer and cell lysates were applied to a single-molecule surface. TIRF images of cell surface receptor proteins pulled-down from native or biotin-labeled cells expressing EGFR-eGFP-flag or eGFP-flag. Scale bar, 5 μm. (**c**) Average numbers of fluorescent molecules per imaging area (*N*_*f*_). Error bars denote standard deviation (*n*>10). Membrane protein biotinylation, MPB.

In previously reported single-molecule isolation techniques^11^,^12^, the host species of surface-tethered antibody to capture interesting proteins makes it difficult to yield multicolor immunofluorescence images. To overcome this, we designed the SiMBlot assay based on cell surface biotinylation^19^,^20^ and single-molecule techniques^21^ (Fig. 1a). Recombinant EGFR (rEGFR) ectopically expressed in mammalian cells (COS7) was tagged with enhanced green fluorescent protein (eGFP) for fluorescence imaging. To specifically immobilize PM-loaded protein molecules from cell extracts onto the single-molecule surface, we labeled only cell surface proteins using an amine-reactive biotin reagent (Sulfo-NHS-Biotin), which is impaired in penetrating diffusion through the cell membrane (Fig. 1a). After cell lysis, crude cell extracts were pulled-down with NeutrAvidin beads or introduced onto a single-molecule surface coated with NeutrAvidin. Only biotinylated cell surface proteins including rEGFR and endogenous IGF-1R, which formerly localized on the cell surface membrane, were unbiasedly isolated by NeutrAvidin beads, not cytosolic proteins such as eGFP (Supplementary Fig. 1), and they were also directly immobilized onto the single-molecule surface by biotin-NeutrAvidin pairing (Fig. 1a,b). Although loading cell extracts containing the same amount of fluorescent protein resulted in identical non-specific absorption onto an uncoated glass surface (Supplementary Fig. 2), only the lysate of membrane-biotinylated cells expressing EGFR-eGFP-flag showed a significantly high amount of eGFP fluorescence signals on the NeutrAvidin-coated glass via specific biotin–NeutrAvidin pairing (Fig. 1c). These results indicate that biotinylated membrane proteins including rEGFR are specifically immobilized with intended molecular density on the single-molecule surface. Therefore, the SiMBlot assay can be used to study the state of RTKs at the single-molecule level, without technical impedance by high molecular density proteins such as EGFR on the cell membrane^17^.

### Detection of EGF-induced receptor tyrosine phosphorylation

**Figure 2:**
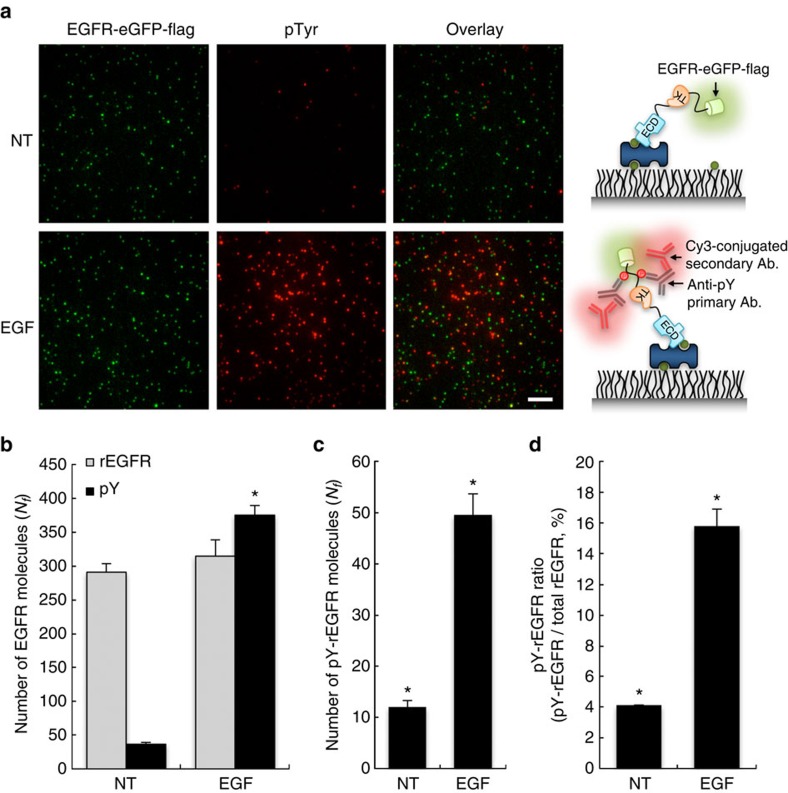
Colocalization analysis of EGF-induced tyrosine phosphorylation and recombinant EGFR. After starvation for 24 h, COS7 cells expressing EGFR-eGFP-flag were labeled with a biotin derivative reagent and incubated with or without EGF (100 ng ml^−1^) for 10 min. Cells were extracted using lysis buffer and cell lysates were applied to a single-molecule surface. (**a**) eGFP signals were from pulled-down EGFR-eGFP-flag. Cy3 signals were from probing tyrosine phosphorylation using a primary antibody against tyrosine phosphorylation and a fluorescently-labeled secondary antibody. Scale bar, 5 μm. (**b**) Average numbers of fluorescent molecules per imaging area (*N*_*f*_). Error bars denote standard deviation (*n*>10). (**c**,**d**) Colocalization numbers and ratios of pTyr on rEGFR. *P<0.05, Student's *t*-test.

Since cell surface biotinylation may affect the physiological function of PM-loaded receptor proteins, we examined the biological function of biotinylated membrane proteins by using conventional western blot analysis for RTK signaling. We observed identical EGF-dependent tyrosine phosphorylations of unlabeled and biotinylated EGFR (Supplementary Fig. 3) as an indicator of EGFR activation, which induces tyrosine autophosphorylation at multiple tyrosine residues in the cytoplasmic domain by an indwelling tyrosine kinase activity^2^. These results show that PM-loaded receptor proteins including EGFR are functional after cell surface biotinylation for the SiMBlot assay. Then, to confirm whether SiMBlot also reflects EGF-induced receptor phosphorylation at the single-molecule level, we immobilized cell surface proteins on the single-molecule surface and probed tyrosine phosphorylation using an anti-pTyr primary antibody and a Cy3-labeled secondary antibody. With SiMBlot, the surface showed a high amount of eGFP fluorescence signals (Fig. 2a). We quantified EGF-dependent activation of EGFR by probing tyrosine phosphorylation of immobilized proteins (Fig. 2b). Relative to the amount of eGFP signals, many Cy3 signals and incomplete colocalization likely arose from defective eGFP maturation^22^ and tyrosine phosphorylations of non-EGFR membrane proteins and especially endogenous EGFR. Indeed, this was reflected in a western blot analysis (Supplementary Fig. 3), as endogenous EGFR (64.8%) was more tyrosine phosphorylated than rEGFR (7%) and non-EGFR cell surface proteins (28.2%). Accordingly, excluding un-colocalized Cy3 signals, we counted and quantified tyrosine phosphorylation of rEGFR using colocalization analysis between eGFP and Cy3 signals (Fig. 2c); the percentage of tyrosine phosphorylated rEGFR was ∼4 and ∼16% in the basal condition and following EGF treatment, respectively (Fig. 2d). These results show that EGF increased tyrosine phosphorylation of rEGFR by 4-fold. These observations provide the exquisite quantitative data for EGF-induced EGFR phosphorylation, avoiding signal amplification by multiple primary and secondary antibody binding. Therefore, SiMBlot can be used to quantitatively analyze ligand-dependent RTK signaling at the single-molecule level.

### Quantitative analysis of site-specific phosphorylations

Moreover, to test whether SiMBlot can reflect the results of a conventional method for site-specific modifications, we measured site-specific phosphorylation of EGFR in response to EGF (100 ng ml^−1^) by SiMBlot and immunoblotting, as an ensemble analysis. As reported previously^23^, EGFR activated by EGF binding could be phosphorylated at Thr669, Tyr845, Tyr1068 and Tyr1173. In the western blot analysis, the tyrosine sites were rapidly phosphorylated at an early time point (about 2–4 min), whereas the Thr669 site was phosphorylated later (10–30 min) (Supplementary Fig. 4a,b). At the same time, we quantified each phosphorylation site of EGFR at various time points using the SiMBlot assay (Supplementary Fig. 4c) with site-specific phospho-antibodies. These antibodies were verified by ectopic expression of wild type (WT) and mutant rEGFR (Y845F, Y1068F and Y1173F) on chinese hamster ovary (CHO) cells, which lack endogenous EGFR (Supplementary Fig. 5). Each antibody is a highly specific probe for the detection of EGFR site-specific auto-phosphorylations in the SiMBlot assay. These results closely resembled the results of the conventional method. Accordingly, SiMBlot can provide the same results as a conventional method based on quantified data at the single-molecule level.

### Multiple immunolabeling on a single polypeptide molecule

In the SiMBlot assay, antibody-free immobilization provides a more flexible choice of antibody for immunolabeling because it is not constrained by the immobilization antibody species. Furthermore, this flexibility can make it easier to prepare antibody sets adequate for simultaneous multiple immunolabeling. On a single polypeptide molecule, the multicolor immunofluorescence labeling can enable to directly probe site-specific combinatorial PTMs of individual polypeptide molecules. Although multiple immunolabeling of a single polypeptide molecule may be hampered by steric hindrance, this possibility can be readily minimized by adding 2% sodium dodecyl sulfate and boiling the cell lysate to denature proteins, as described in the Methods section. Denaturation to the unfolded linear shape can make a space sufficient for an antibody binding site (over 15 amino acids (>5 nm)ref. 24), binding of a large-sized antibody, and multiple associations of the secondary antibody with the primary antibody to evade steric hindrance by separating multiple modification sites as spatially far away from each other as possible. Therefore, protein denaturation should be remarkably helpful to analyze site-specific PTM patterns within individual protein molecules.

### EGF-induced EGFR phosphorylation pattern on cell membrane

**Figure 3:**
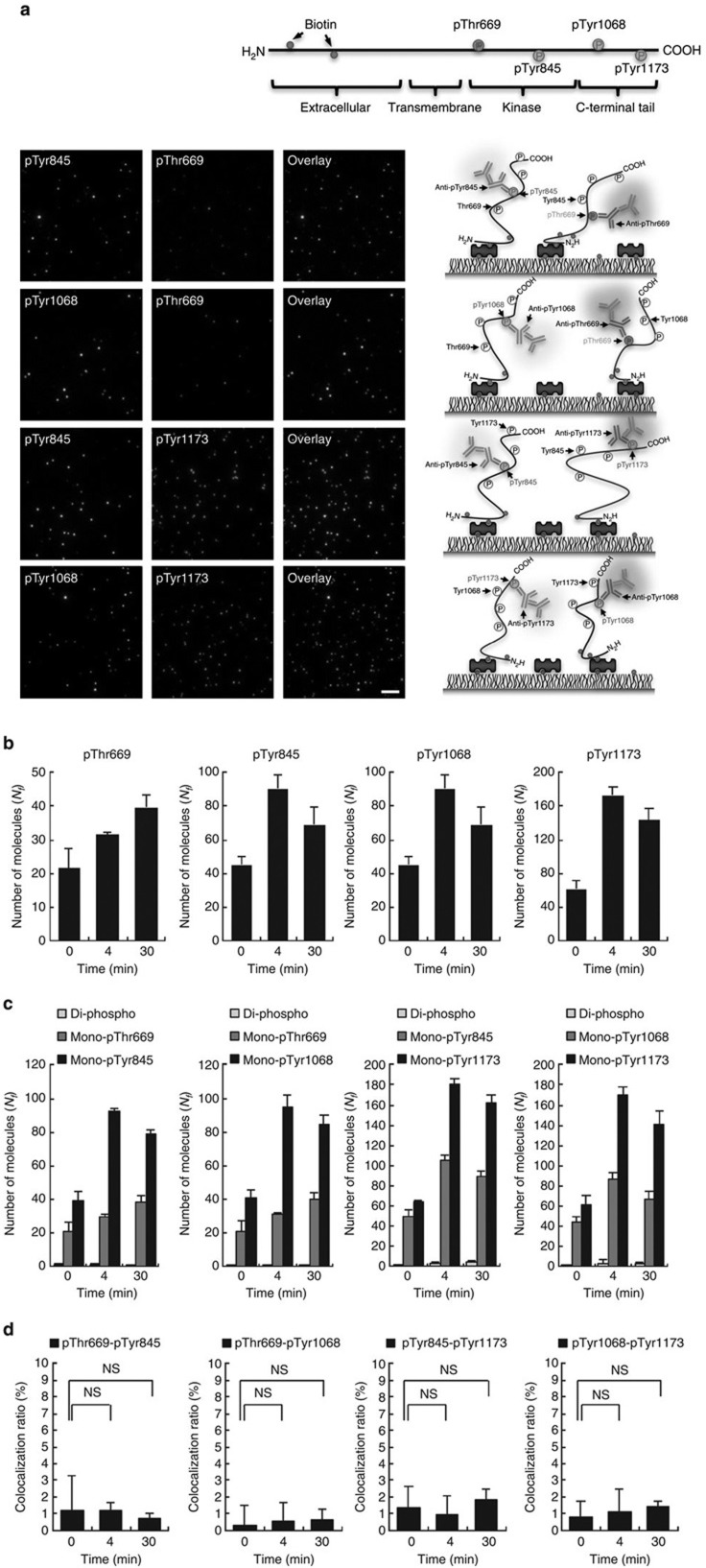
EGF-induced pairwise phosphorylation pattern of EGFR on A431 cells. After starvation for 24 h, A431 cells were labeled with a biotin derivative reagent and incubated with EGF (100 ng ml^−1^) for the indicated amount of time (0, 4, and 30 min). Denatured whole-cell lysates were prepared and subjected to SiMBlot with the indicated site-specific phosphorylation antibody sets (pTyr1068/pTyr1173, pThr669/pTyr1068, pTyr845/pTyr1173 and pThr669/pTyr845). (**a**) After incubation for 30 min with EGF, representative SiMBlot images of Alexa488 and Cy3 signals generated by probing site-specific phosphorylation with the indicated primary and fluorescently-labeled secondary antibodies. Scale bar, 5 μm. (**b**,**c**) Graphs show the average numbers of fluorescent molecules per imaging area (*N*_*f*_) using the indicated site-specific phosphorylation antibodies. (**d**) The EGF-induced pairwise phosphorylation pattern of individual endogenous EGFR molecules was analyzed by colocalization of the indicated antibody sets. Error bars denote standard deviation (*n*>5). NS, not significant.

To confirm the ability of SiMBlot to directly visualize pairwise site-specific modifications of individual proteins, we used EGF-dependent phosphorylated EGFR. Since EGF-induced multiple phosphorylation of EGFR has been suggested by enormous ensemble biochemical results and it has long been widely believed^1^,^2^,^18^, it should be possible to detect multi-phosphorylation patterns of individual EGFR molecules by SiMBlot. We therefore performed the SiMBlot assay with colocalization analysis of site-specific phosphorylations of EGF-induced endogenous EGFR taken from A431 cells using antibody sets to detect pairwise site-specific phosphorylation; the autophosphorylation sets (pTyr845 and pTyr1068 or pTyr1173 of EGFR) and the feedback sets (pThr669 and pTyr845 or pTyr1068 of EGFR). Each Alexa488 and Cy3 fluorescence signal indicated site-specific phosphorylation (Fig. 3a,b), and colocalization demonstrated di-phosphorylation in individual EGFR molecules (Fig. 3a,b). Strikingly, colocalization was rarely detected, regardless of the antibody pair (Fig. 3c); ≤2% of total fluorescence signals colocalized and each colocalization ratio did not significantly change over time (Fig. 3d), although the number of fluorescence signals reflecting each phosphorylation site independently increased in a time-dependent manner (Fig. 3b). This unappreciated information can either be not provided or overlooked by conventional biochemical methods (Supplementary Fig. 6), which do not probe the phosphorylation pattern in individual EGFR molecules. Moreover, these results demonstrate that multiple EGF-dependent EGFR autophosphorylation events do not coincide with negative feedback phosphorylation or with each other. Making an objection to the multi-phosphorylation model, these findings may propagate to redefine the negative feedback role of Thr669 (ref. 25) as the desensitizing signal, and the importance of intermolecular collaboration among multiple autophosphorylated EGFR molecules rather than intramolecular cooperation among multiple autophosphorylated sites in a single EGFR molecule^26^.

### Phosphorylation pattern of *in vitro* autophosphorylated EGFR

**Figure 4:**
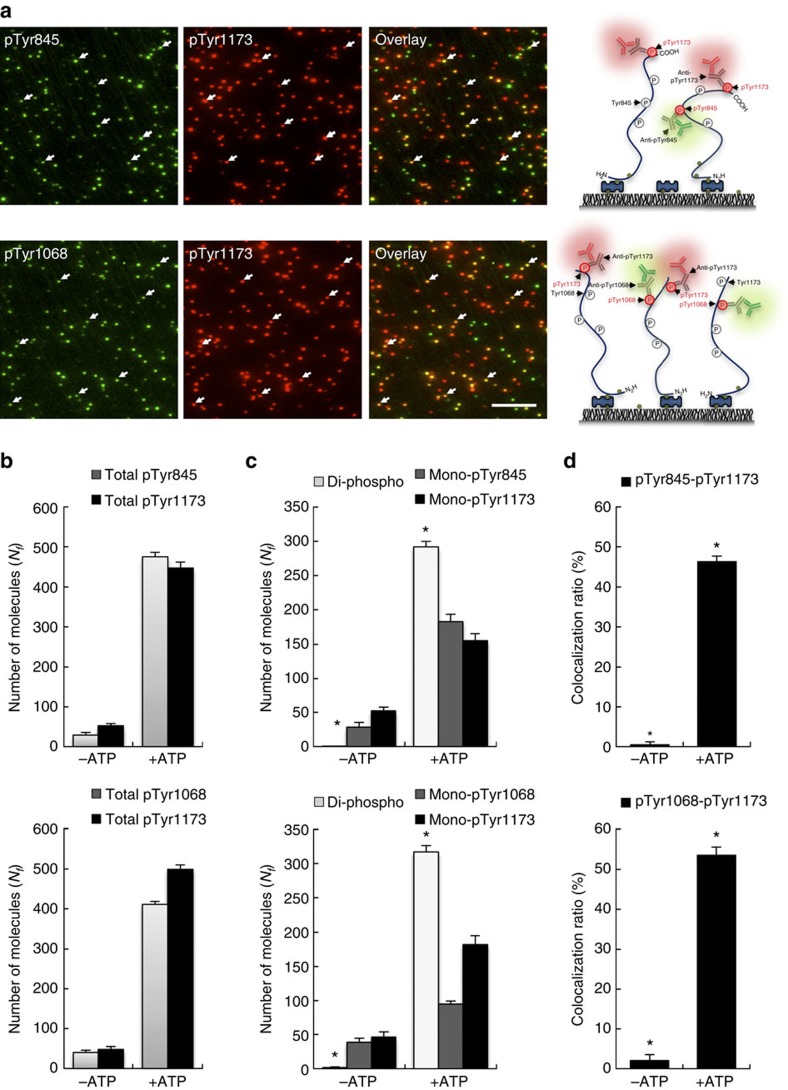
Detection of pairwise site-specific phosphorylation of *in vitro* autophosphorylated EGFR. After starvation for 24 h, COS7 cells expressing EGFR-eGFP-flag were labeled with a biotin derivative reagent and extracted using lysis buffer containing 10 μM AG1478. For *in vitro* autophosphorylation, affinity-purified rEGFR was incubated with 100 ng ml^−1^ EGF and 100 μM ATP for 1 h, as described in the Methods section. Autophosphorylation sites of rEGFR were quantified by the SiMBlot assay with the indicated antibody sets. (**a**) Representative SiMBlot images of Alexa488 and Cy3 signals generated by probing site-specific phosphorylation with the indicated primary and fluorescently-labeled secondary antibodies. Scale bar, 5 μm. (**b)** Graphs show the average numbers of fluorescent molecules per imaging area (*N*_*f*_) using the indicated antibodies. (**c**) Pairwise phosphorylation of EGFR was analyzed by colocalization of the indicated antibody sets. (**d**) Colocalization ratio of the indicated antibody sets. Error bars denote standard deviation (*n*>5). *P<0.05, Student's *t*-test.

To examine whether the low level of colocalization in the cell model experiment is due to steric hindrance induced by simultaneous multiple immunolabeling, we prepared purified autophosphorylated rEGFR molecules by affinity purification and performed an *in vitro* rEGFR kinase assay to phosphorylate most tyrosine residues in its C-terminal tail, as evidenced by the detection of autophosphorylation by immunoblotting (Supplementary Fig. 7). Then, we performed colocalization analysis of *in vitro* autophosphorylated rEGFR with pairwise autophosphorylation antibody sets (pTyr845/pTyr1173 and pTyr1068/pTyr1173). After immobilization of rEGFR (Supplementary Fig. 8), we probed each autophosphorylation of rEGFR with the pairwise antibody sets (Fig. 4a). The antibody specificity was validated by using synthetic phospho-peptides (pY845, pY1068 and pY1173; Supplementary Fig. 9) and rEGFR mutants (Y845F, Y1068F and Y1173F; Supplementary Fig. 10). Upon *in vitro* autophosphorylation, the numbers of autophosphorylated rEGFR molecules increased by ∼10-fold (Fig. 4b). As expected, unlike the cell model experiment (Fig. 3c), colocalization of Alexa488 and Cy3 fluorescence signals significantly increased in *in vitro* autophosphorylated rEGFR (Fig. 4c). In the autophosphorylation pairs, nearly 50% of total fluorescence signals significantly colocalized, which indicated di-phosphorylated rEGFR (Fig. 4d). Moreover, the comparative colocalization ratio of autophosphorylation pairs (pTyr845/pTyr1173 and pTyr1068/pTyr1173) reached nearly 70% (Supplementary Fig. 11). Furthermore, comparable with the number of fluorescence signals in the A431 cell experiment, diluted autophosphorylated rEGFR even had high colocalization ratios (pTyr845/pTyr1173: 35.4% and pTyr1068/pTyr1173: 42.4%) of the autophosphorylated pairs (Supplementary Fig. 12), despite a small decrease in the colocalization ratios, which may arise from the difference in the influence of background signals.

These results confirm that individual multi-autophosphorylated EGFR can be simultaneously labeled by the pairwise autophosphorylation antibody sets without any hindrance. At the same time, the unexpected site-specific phosphorylation patterns of EGF-induced EGFR on the cell membrane (Fig. 3c,d), which had been veiled in ensemble results so far, can also be verified. Therefore, these data show the distinctive ability of our method in single-molecule studies of PTMs, which can readily visualize unprecedented pairwise site-specific modifications within individual polypeptide molecules. However, as evaluated by the western blot analysis with the same samples (Supplementary Figs 6,7), the discrimination between a mixture of mutually exclusively mono-phosphorylated EGFR molecules and simultaneously multi-phosphorylated EGFR molecules is too difficult to be achieved using conventional ensemble methods, which have unavoidable limitations in analyzing pairwise site-specific phosphorylation^6^.

### Co-occurrence of pairwise autophosphorylation sites

We also examined the relationship between the pairwise autophosphorylation sites of *in vitro* autophosphorylated EGFR. The simultaneous multi-autophosphorylations (Fig. 4c,d) may come from the closely interrelated autophosphorylations between each site or the continuous accumulations of random autophosphorylations. To characterize the multi-autophosphorylations, we tested the co-occurrence of the pairwise autophosphorylation sites by using *Lift* value on association rule mining^27^,^28^, which is the ratio of the observed frequency of co-occurrence to the expected frequency. In statistics, *Lift* value is simply estimated by the ratio of the comparative colocalization ratio (used for P(x|y); Supplementary Fig. 11) divided by the individual autophosphorylation ratio (used for P(x); Supplementary Fig. 13) on total rEGFR numbers: Lift(x,y)=P(x|y)/P(x)=P(y|x)/P(y). Because the phospho-antibodies have an incomplete site-specificity (Supplementary Table 1), each probability was corrected by the respective site-specificity of the phospho-antibodies (Supplementary Table 2). Intriguingly, the obtained *Lift* values (pTyr845/pTyr1173: 0.91–0.93, pTyr1068/pTyr1173: 1.13–1.14) are approximately equal to 1 (Supplementary Table 3), although the trivial differences between P(x|y)/P(x) and P(y|x)/P(y) values are likely derived from measurement error or steric hindrance. These results would imply that the autophosphorylation event of each tyrosine residue is most likely independent of each other and thus the multi-autophosphorylation is a result of the repeated random autophosphorylations. From the independency between autophosphorylation sites, we conjecture that EGFR kinase activity has no inherent selectivity to recognize prior autophosphorylation of EGFR, explaining why EGF-induced EGFR on cell membrane was mostly mono-phosphorylated and rarely multi-phosphorylated (Fig. 3). Moreover, combined with EGF-dependent EGFR clustering on the cell membrane^29^, our observations suggest that each site-specific phosphorylated EGFR likely has individual specific roles to recruit specific molecules for downstream signaling and to participate in receptor clustering and complex formation for EGFR signaling, suggesting why EGFR should oligomerize for the signaling complex. A more in-depth examination of the mechanistic model of EGFR activation (Supplementary Fig. 14) will be reported elsewhere.

## Discussion

Here we describe a simple and versatile method to monitor pairwise site-specific PTM patterns of RTKs at the single-molecule level. Like previous single-molecule isolation techniques, SiMBlot can be also used with conventional western blot analysis and take about 30 min, considerably shorter than conventional ensemble methods. In particular, SiMBlot can readily provide quantitative data of the PTM status of individual RTK molecules on different conditions without any signal amplification by multiple associations of secondary antibodies with the primary antibody and multiple enzyme-labeled antibodies like in western blot analysis. Also, its antibody-free immobilization confers distinctively a more flexible choice on commercially available antibodies for the detection of site-specific PTMs. Moreover, the colocalization analysis of the multicolor immunofluorescence labeling in the SiMBlot assay enables to directly visualize heterogeneous PTM isoforms with unique combinations of multiple PTM sites. Indeed, we observed unprecedented single-molecule pairwise site-specific phosphorylation patterns of EGF-induced or *in vitro* autophosphorylated EGFR (Figs 3 and 4) by the colocalization analysis, although multiple labeling in a short sequence (<100 amino acids, not tested) still remains uncertain. These were made possible by our blotting method, which ensures flexible antibody choice, minimal steric hindrance and exquisite qualitative and quantitative analysis without ensemble error.

Combinatorial PTMs on a single polypeptide molecule garner inevitable interest^9^,^10^ but remain an unexplored area. In the last decade, MS, which is popularly used to analyze the proteome, has become a powerful technology to identify diverse PTMs^5^ and measure the heterogeneity of proteoforms^30^,^31^. In comparison with SiMBlot, which can faithfully analyze even widely spaced phosphorylation sites at the single-molecule level (even if >300 amino acids), MS-based proteomics are limited by the trade-off between ‘bottom-up' proteomics for the identification of site-specific PTMs of digested peptides (10∼20 amino acids) and ‘top-down' proteomics for the analysis of the proteoform heterogeneity of intact proteins^8^,^31^. In addition, MS-based proteomics necessitates effective fractionation with extensive additional sample preparation and an expensive high-resolution mass spectrometry^30^. Although MS-based proteomics has several limitations for the analysis of combinatorial PTMs^32^, it is still valuable for both large-scale and targeted approaches to provide a ‘bird's eye' view of the proteoforms and relative abundances^31^. Both SiMBlot and MS-based proteomics have their differential advantages and disadvantages, and thus can complement each other to precisely detect co-occurrence of pairwise site-specific modifications at the single-molecule level, which will help to elucidate the post-translational logic of intracellular signaling.

Besides MS-based proteomics, recently, the nanopore and nanogap methods in nanotechnology are rapidly developing but remain incompatible for single-molecule proteomic analysis of PTMs^13^,^14^. These approaches can only assess *in vitro* modified proteins or peptides, not those acquired from cells, and analyze a confined short segment (about 20 amino acids from the N or C terminus) of the polypeptide chain to be examined. However, SiMBlot can be easily applied to analyze even the endogenously modified proteins taken from cells by probing for PTM sites dispersed along the entire protein length using available site-specific primary antibody and fluorescently labeled secondary antibody.

Although we only report the application of this method to combinatorial PTMs of EGFR, previous studies^20^,^33^,^34^ suggest that our methods should be compatible with other cell surface receptors, supposing that appropriate site-specific modification antibody is available. Along with continuous development of site-specific PTM antibody, SiMBlot would be more versatile and robust method for clarification and better understanding of the recondite pairwise site-specific PTM patterns of PM receptors at the single-molecule level. Furthermore, the advent of selective biotinylation of specific residues or tags by chemical^35^ or enzymatic^36^ means and the development of genetic engineering techniques should extend the application of SiMBlot to various unexplored signaling proteins containing multiple PTM sites, such as the C-terminal domain of polymerase and histone codes^1^,^37^.

## Methods

### Antibodies and reagents

Antibodies against EGFR, phospho-EGFR (pThr669, pTyr1068 and pTyr1173), and phospho-Erk were purchased from Cell Signaling Technology (Cat. #2239, #3056, #2236, #4407 and #9101). A monoclonal anti-pTyr845 EGFR antibody was purchased from Millipore (Cat. #04-283). A monoclonal anti-β-actin antibody was purchased from MP Biomedicals (Cat. #08691001). An Alexa488-labeled antibody and Lipofectamine were purchased from Invitrogen Life Technology (Cat. #A110011, #18324). A Cy3-labeled secondary antibody was purchased from Jackson ImmunoResearch Inc. (Cat. #111-165-003). Recombinant human EGF was purchased from R&D Systems (Cat. #236-EG). Sulfo-NHS-Biotin was purchased from Thermo Scientific (Cat. #21217). Anti-FLAG M2 affinity gel, 3 × FLAG peptides, and Phosphatase Inhibitor Cocktail 3 were purchased from Sigma-Aldrich (Cat. #A2220 and #F4799). Phosphopeptides (pY845: AKLLGAEEKEpYHAEGGKVPI, pY1068: DDTFLPVPEpYINQSVPKRPA and pY1173: KGSTAENAEpYLRVAPQSSEF) were purchased from PEPTRON Inc.

### Cell culture and transfection

COS7 and A431 cells were maintained in Dulbecco's Modified Eagle's Medium supplemented with 10% (v/v) fetal bovine serum at 37 °C in a humidified CO_2_-controlled (5%) incubator. CHO-K1 cells were maintained in Ham's F-12K medium supplemented with 10% (v/v) fetal bovine serum at 37 °C in a humidified CO_2_-controlled (5%) incubator. For transfection and transient expression of proteins, cells were transfected with plasmids encoding rEGFR using Lipofectamine (Invitrogen, Carlsbad, CA). Transfections were performed using Lipofectamine (according to the manufacturer's instructions) and cells were then cultured for an additional 48 h to achieve ectopic expression of rEGFR.

### Plasmids

A plasmid encoding C-terminal flag-tagged eGFP was constructed by insertion of the corresponding cDNA sequences of eGFP into the XbaI/AgeI sites of the pcDNA 3.1 myc/his plasmid (Invitrogen). The inserted DNA fragment encoding C-terminal flag-tagged eGFP was prepared by PCR using the pEGFP-C1 vector (Clontech Laboratory Inc.) as the template and primers (forward: 5′- GCTCTAGA GGAGGG ATGGTGAGCAAGGGCGAGGAG -3′, reverse: 5′- C ACCGGT TCA CTTGTCGTCATCGTCTTTGTAGTC CTTGTACAGCTCGTC -3′). The constructs for WT and mutant rEGFR (Y845F, Y1068F and Y1173F) were cloned by inserting the N-terminus of the corresponding cDNA sequence of whole EGFR into pcDNA3.1 eGFP-flag using EcoRI/XbaI without its own stop codon.

### Cell surface biotinylation and preparation of cell lysates

Cell surface labeling was conducted as previously described, with minor modifications^38^. COS7 and A431 cells were cultured to 70–80% confluency before addition of a ligand such as EGF. Cells were briefly washed with ice-cold phosphate-buffered saline (PBS), and then 0.5 mg ml^−1^ Sulfo-NHS-Biotin (Thermo Scientific, Cat. #20217) prepared in PBS was added. Cells were incubated with gentle agitation for 20 min at 4 °C and further incubated with 0.1 M glycine prepared in PBS for 10 min at 4 °C. After washing with serum-free Dulbecco's Modified Eagle's Medium, cells were treated with a ligand such as EGF at 37 °C for 10 min, the medium was removed, and cells were washed with ice-cold PBS. Cells were lysed in lysis buffer (50 mM Tris-HCl, pH 7.4, 150 mM NaCl, 5 mM MgCl_2_, 1 mM EDTA, 5% glycerol, 1% NP-40, and a phosphatase inhibitor cocktail) by sonication and cell lysates were centrifuged at 13,500 rpm, 4 °C for 10 min. Supernatants were prepared for single-molecule imaging or subjected to sodium dodecyl sulfate (SDS)-polyacrylamide gel electrophoresis and immunoblotted.

### *In vitro* EGFR kinase assay

COS7 cells ectopically expressing rEGFR were serum-starved for 12 h. After cell surface biotinylation and cell lysate preparation using lysis buffer (containing 10 μM AG1478, an EGFR kinase inhibitor for preservation of the phosphorylation status in living cells (Supplementary Fig. 15)), rEGFR was affinity-purified using anti-FLAG M2-conjugated agarose, followed by washing three times with lysis buffer (AG1478-free) and then two times with kinase buffer (25 mM HEPES, pH 7.4, 20 mM MgCl_2_, 5 mM β-glycerophosphate, 0.5 mM dithiothreitol and 0.1 mM sodium orthovanadate). Immunoprecipitates were eluted from beads with 0.1 mg ml^−1^ 3 × FLAG peptide at 4 °C for 30 min. Purified rEGFR was incubated for 1 h at 30 °C with shaking. EGFR was activated by adding 0.1 mM ATP and 100 ng ml^−1^ EGF prepared in kinase buffer. The reaction was stopped by adding 2% SDS. Samples were boiled for 10 min and subjected to single-molecule imaging or immunoblotting.

### Flow chambers and single-molecule immobilization

Extensively cleaned cover glasses were prepared by cleaning with H_2_O and 1 M KOH for 2 h or longer, and treated with 3-(2-aminoethylamino)-propyltrimethoxysilane (Tokyo Chemical Industry Co., Cat. #A0774) and doped with mPEG and biotin-PEG (Laysan Bio, Inc., mPEG-SVA-5000 and Biotin-PEG-SVA-5000) with the mass ratio of 25:1 (mPEG:biotin-PEG)^39^. Then the passivated cover glasses were coated with 25 mM MS(PEG)4 (Thermo Scientific, Cat. #22341) once more. Flow chambers assembled with the passivated cover glass and slide, and coated with 0.1 mg ml^−1^ NeutrAvidin (Thermo Scientific, Cat. #31000) ref. 39.

For pairwise antibody probing, samples were denatured by boiling for 5 min with 2% SDS. Samples were serially diluted to obtain well-isolated spots on the NeutrAvidin-coated surface upon incubation for 10 min. All dilutions were made immediately before experiments with buffer SB18 (40 mM HEPES, 105 mM NaCl, 5 mM KCl, 5 mM MgCl_2_, 0.05% (v/v) Tween-20 and 0.1 mg ml^−1^ bovine serum albumin, pH 8.0). Unbound antibodies and samples were removed from the channels by washing two times with buffer SB18. For immunofluorescence detection, immobilized protein molecules were incubated with a different antibody (13.3 nM) against the prey protein for 10 min and with a fluorescent dye-labeled secondary antibody (2.66 nM) for 10 min immediately before imaging.

### Single-molecule imaging and analysis

An objective-type TIRF microscope (Olympus IX71, Andor EMCCD) was used to acquire single-molecule data^11^. eGFP and Alexa488 were excited at 488 nm, and Cy3 was excited at 532 nm. Narrow band-pass filters were used to avoid crosstalk between channels (Semrock for eGFP and Alexa488 and Chroma for Cy3). All experiments were performed at room temperature unless otherwise specified. Single-molecule analysis was performed as previously described^40^. The mean spot count per image (imaging area, 2,500 μm^2^) and standard deviation were calculated from images of five or more different regions.

Colocalization between Alexa488 and Cy3 was assessed as described previously, with minor modifications^11^. Briefly, two separate movies of the same region were taken using Alexa488 and Cy3 excitation. The fluorescent spots in both images were fitted with Gaussian profiles to determine the center positions of the molecules to half-pixel accuracy. Next, for each molecule in the Alexa488 image, Cy3 molecules whose center was within a distance of three pixels (∼300 nm) were determined. The number of colocalized molecules divided by the total number of Alexa488 and Cy3 signals was presented as the colocalization ratio. The number of colocalized molecules divided by the number of Alexa488 or Cy3 signals was presented as the comparative colocalized ratio.

### Statistical analysis

All data are presented as the mean±s.d. or representative images of at least three sets of independent experiments. When necessary, data were statistically analyzed using the Student's *t*-test.

## Additional information

**How to cite this article:** Kim, K. L. *et al*. Pairwise detection of site-specific receptor phosphorylations using single-molecule blotting. *Nat. Commun.* 7:11107 doi: 10.1038/ncomms11107 (2016).

## Supplementary Material

Supplementary Information Supplementary Figures 1-15 and Supplementary Tables 1-3
