# Supplementary material for: Pairwise detection of site-specific receptor phosphorylations using single-molecule blotting
Source: Nat Commun. 2016 Mar 24;7:11107. doi: 10.1038/ncomms11107 (PMC4820850; doi:10.1038/ncomms11107)
Supplement: Supplementary Information — Supplementary Figures 1-15 and Supplementary Tables 1-3 [file ncomms11107-s1.pdf]

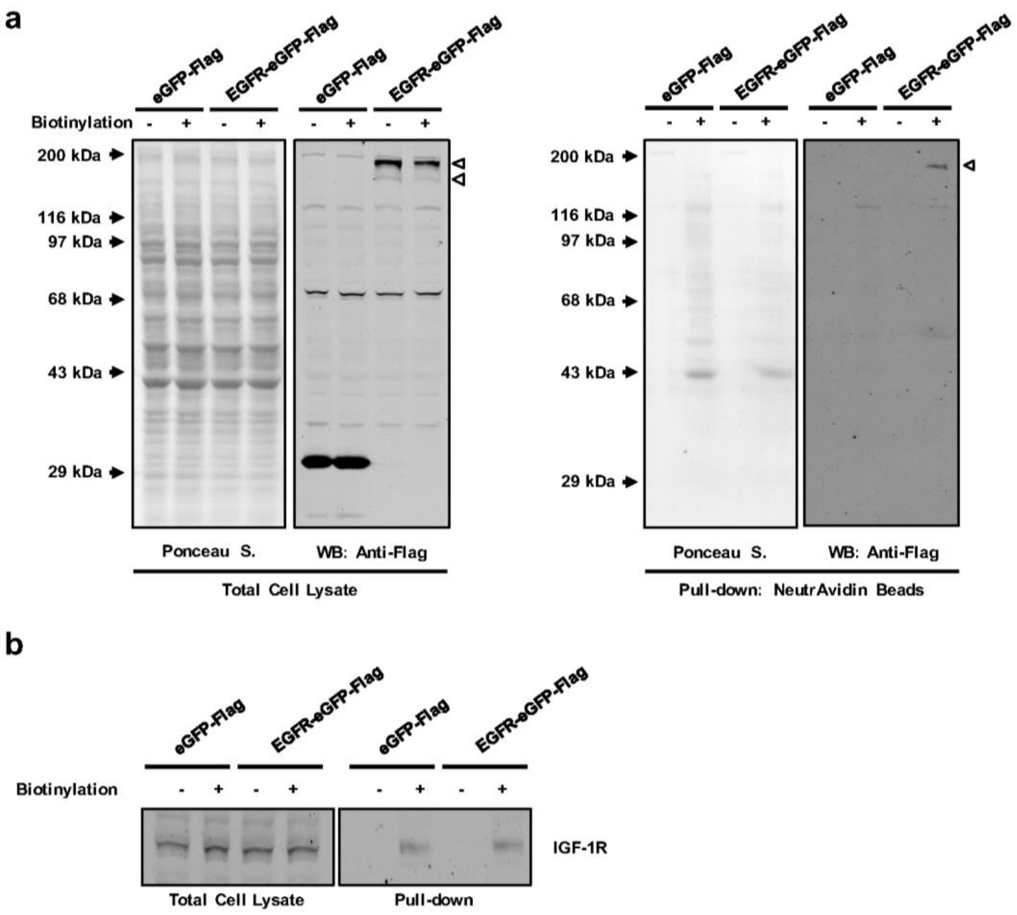


**Supplementary Figure 1. Membrane protein isolation**

COS7 cells expressing EGFR-eGFP-Flag or eGFP-Flag were labeled with a biotin derivative reagent (Sulfo-NHS-Biotin) and lysed with lysis buffer. After incubation of NeutrAvidin beads with the lysate of native or biotin-labeled cells, captured proteins were recovered from the beads by heating in SDS-PAGE sample buffer at 95°C for 5 min. Proteins were resolved by SDS-PAGE and immunoblotted with anti-Flag (**a**) and anti-IGF-1R (**b**) antibodies. The results shown are representative of three independent experiments. Arrowheads indicate EGFR-eGFP-Flag.

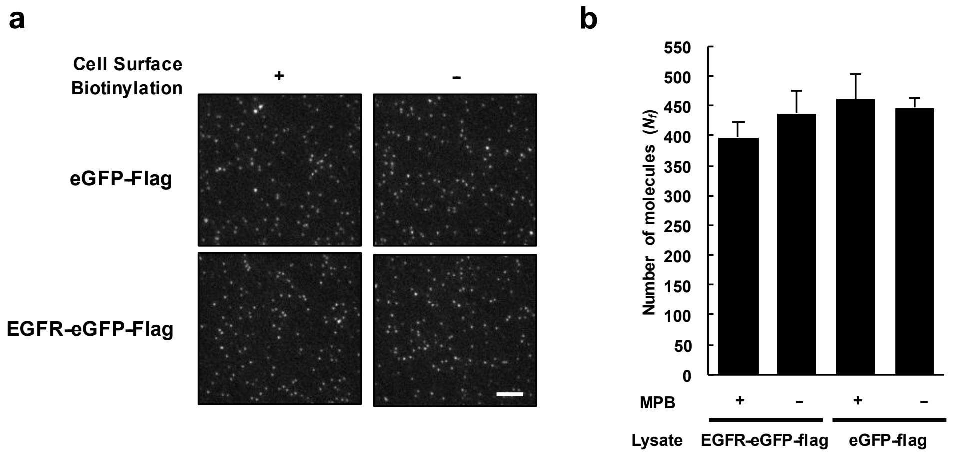


**Supplementary Figure 2. Non-specific absorption of recombinant eGFP proteins on an uncoated glass surface**

(**a**) COS7 cells expressing EGFR-eGFP-Flag or eGFP-Flag were labeled with a biotin derivative reagent and lysed with lysis buffer. Cell lysates were applied to an uncoated glass surface. TIRF images of non-specifically absorbed cell surface receptor proteins from native or biotin-labeled cells expressing EGFR-eGFP-Flag or eGFP-Flag. Scale bar, 5 μm. (**b**) Average numbers of fluorescent molecules per imaging area ($N_f$). Error bars denote standard deviation (n > 10). Membrane protein biotinylation, MPB.

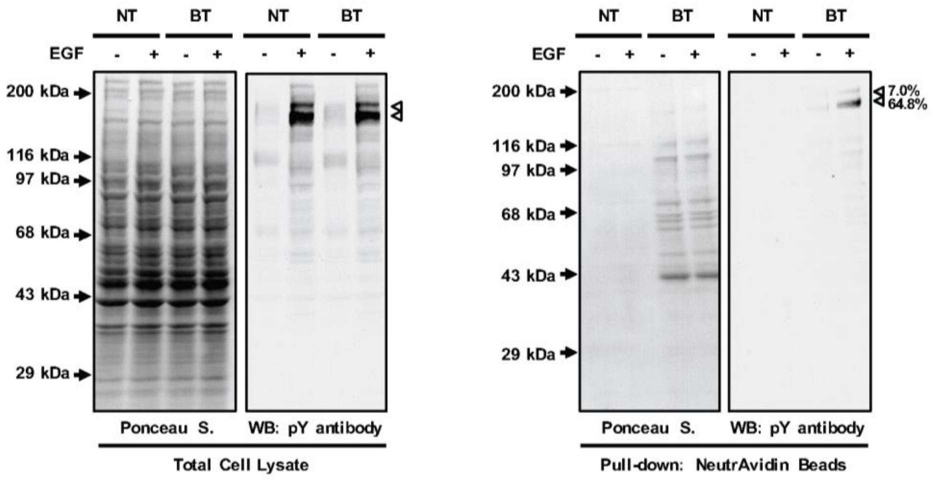


**Supplementary Figure 3. EGF-induced membrane protein isolation**

After starvation for 24 h, COS7 cells expressing EGFR-eGFP-Flag were labeled with a biotin derivative reagent and incubated with or without EGF (100 ng $ml^{-1}$) for 10 min. Experiments were performed in parallel to those shown in Supplementary Figure 1. Proteins were resolved by SDS-PAGE and immunoblotted with an anti-pTyr antibody (left: total cell lysate, right: captured proteins). NT, no treatment. BT, labeled with biotin. Arrowheads indicate EGFR-eGFP-Flag and endogenous EGFR.

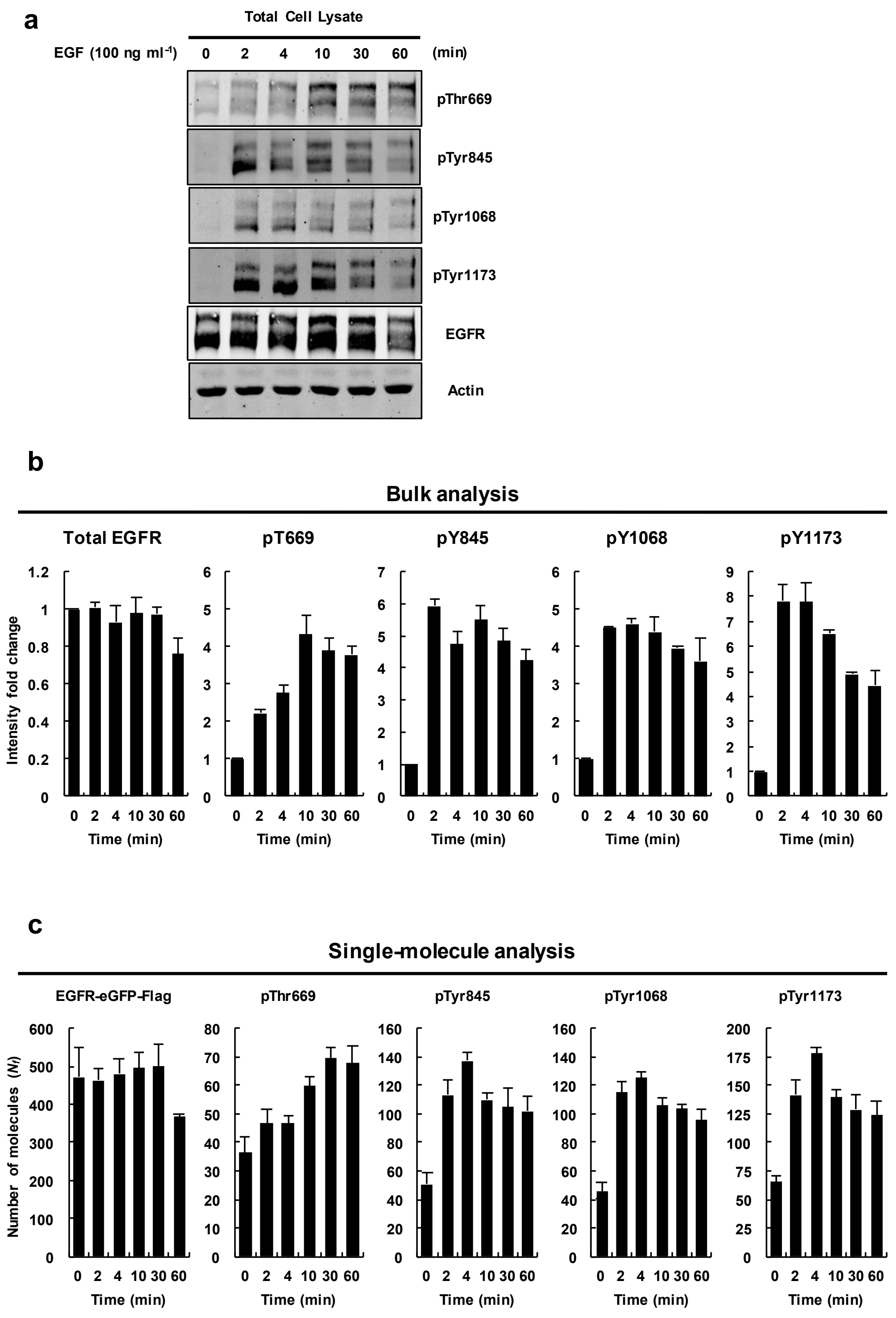


**Supplementary Figure 4. EGF-induced site-specific phosphorylation of EGFR molecules**

After starvation for 24 h, COS7 cells expressing EGFR-eGFP-Flag were labeled with a biotin derivative reagent and incubated with or without EGF (100 ng ml$^{-1}$) for the indicated amount of time (0, 2, 4, 10, 30, and 60 min). Subsequently,

whole-cell lysates (20 µg for immunoblotting and 10 ng for SiMBlot) were prepared and subjected to immunoblotting (**a, b**) and SiMBlot (**c**) with the indicated antibodies (primary antibody: 1 ng $ml^{-1}$ for immunoblotting and 2 ng $ml^{-1}$ for SiMBlot; secondary antibody: 0.2 ng $ml^{-1}$ for immunoblotting and 0.4 ng $ml^{-1}$ for SiMBlot). The results shown are representative of three independent experiments. Graphs show the relative band intensities quantified by densitometry and the average numbers of fluorescent molecules per imaging area ($N_f$) using the indicated antibodies. Error bars denote standard deviation ($n > 5$).

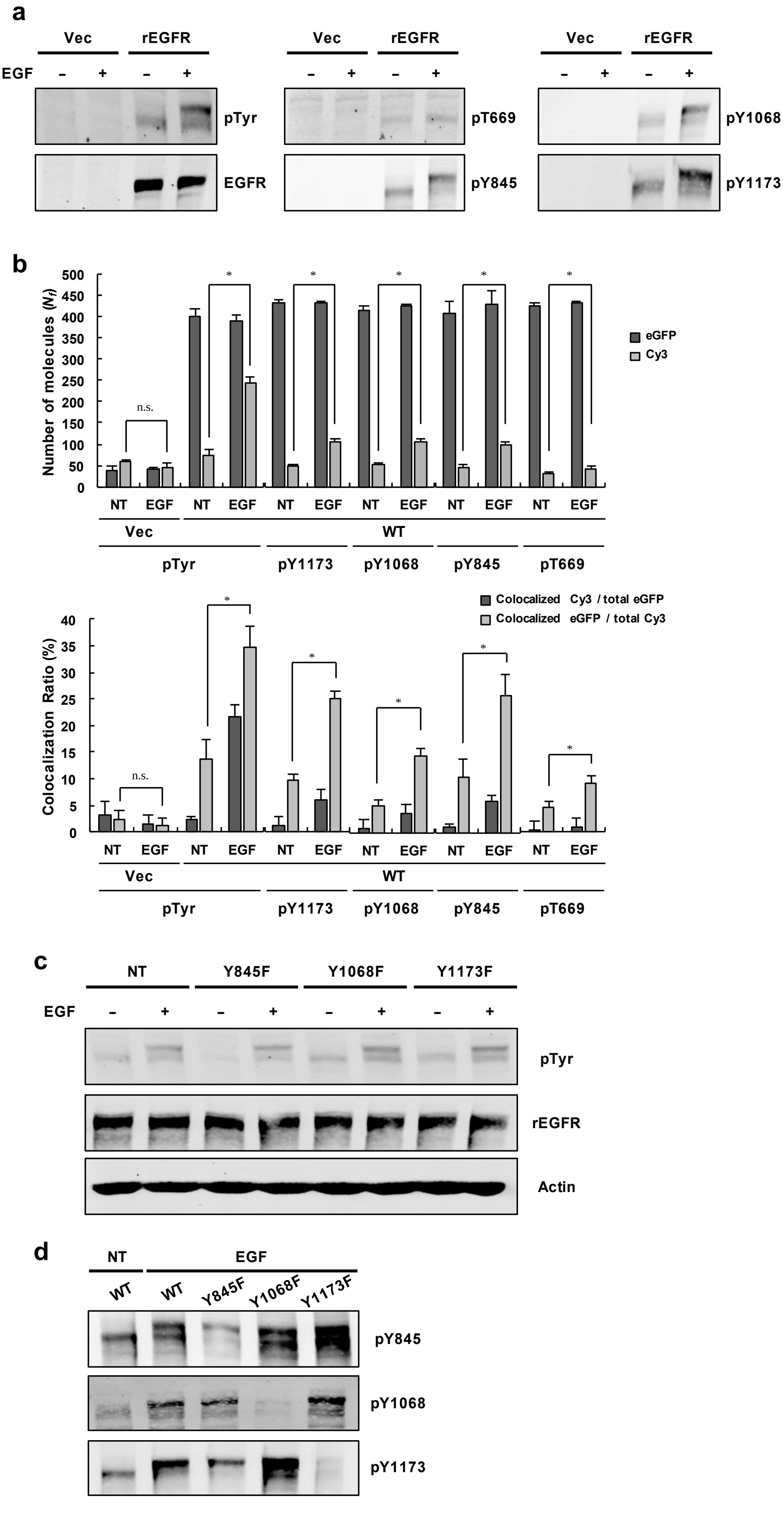
a
Vec
rEGFR
EGF
pTyr
EGFR
pT669
pY845
pY1068
pY1173
b
Number of molecules ($N_f$)
eGFP
Cy3
n.s.
NT
EGF
Vec
WT
pTyr
pY1173
pY1068
pY845
pT669
Colocalized Cy3 / total eGFP
Colocalized eGFP / total Cy3
Colocalization Ratio (%)
c
NT
Y845F
Y1068F
Y1173F
pTyr
rEGFR
Actin
d
WT
pY845
pY1068
pY1173

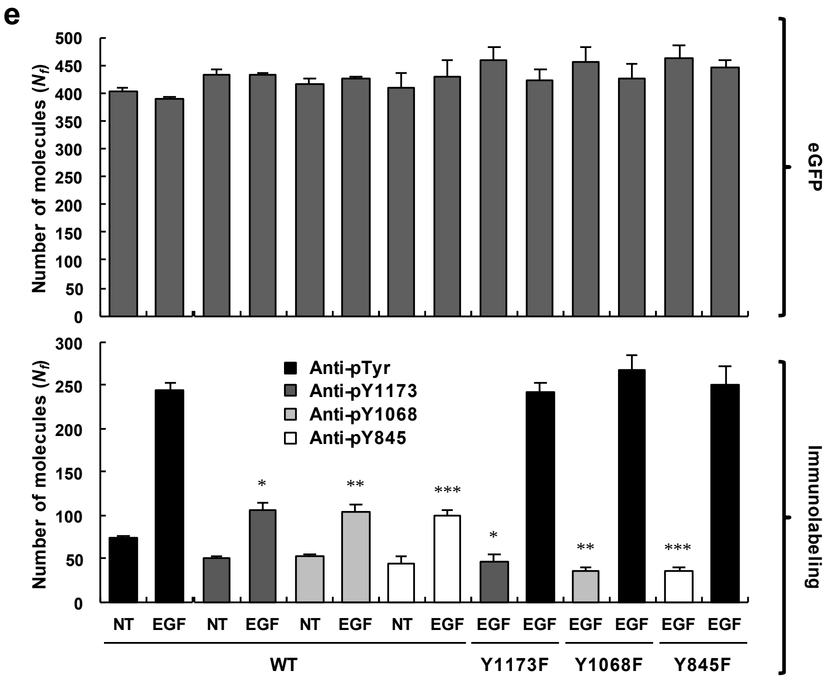


**Supplementary Figure 5. Specific detection by site-specific phospho-antibodies**

After starvation for 24 h, CHO cells expressing vector or EGFR-eGFP-Flag were labeled with a biotin derivative reagent and incubated with or without EGF (100 ng $ml^{-1}$) for 10 min. Subsequently, whole-cell lysates (20 µg for immunoblotting and 10 ng for SiMBlot) were prepared and subjected to immunoblotting (**a**) and SiMBlot (**b**) with the indicated antibodies. After starvation for 24 h, CHO cells expressing WT or mutant EGFR-eGFP-Flag (Y845F, Y1068F, and Y1173F) were labeled with a biotin derivative reagent and incubated with or without EGF (100 ng $ml^{-1}$) for 10 min. Subsequently, whole-cell lysates (20 µg for immunoblotting and 10 ng for SiMBlot) were prepared and subjected to immunoblotting (**c, d**) and SiMBlot (**e**) with the indicated antibodies. The results shown are representative of three independent experiments. Graphs show the relative band intensities quantified by densitometry and the average numbers of fluorescent molecules per imaging area ($N_f$) using the indicated antibodies. Error bars denote standard deviation (n > 5). n.s., not significant. *, **, ***, $p < 0.05$.

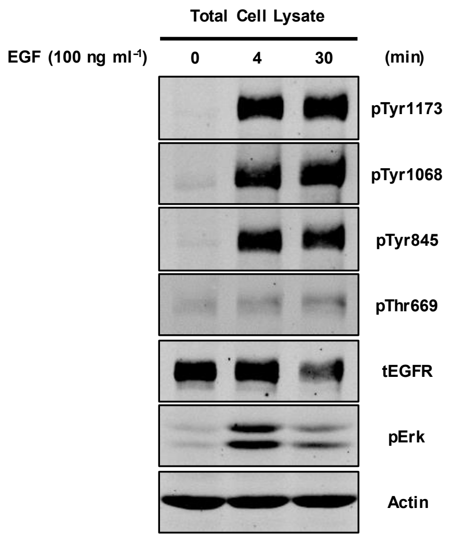


**Supplementary Figure 6. EGF-dependent site-specific phosphorylation of EGFR on A431 cells**

After starvation for 24 h, A431 cells were labeled with a biotin derivative reagent and incubated with EGF (100 ng $ml^{-1}$) for the indicated amount of time (0, 4, and 30 min). Subsequently, denatured whole-cell lysates were prepared and subjected to immunoblotting with the indicated site-specific phosphorylation antibody (pThr669, pTyr845, pTyr1068, and pTyr1173). The result shown is representative of three independent experiments.

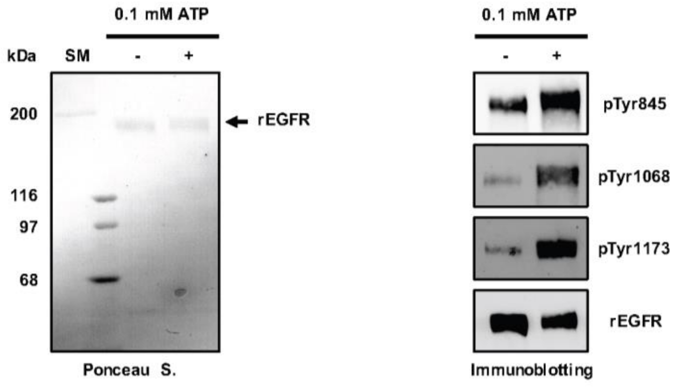


**Supplementary Figure 7. Detection of *in vitro* autophosphorylation of rEGFR by immunoblotting**

After starvation for 24 h, COS7 cells expressing EGFR-GFP-Flag were labeled with a biotin derivative reagent and extracted with lysis buffer. For *in vitro* autophosphorylation, affinity-purified rEGFR was incubated with 100 ng $ml^{-1}$ EGF and 0.1 mM ATP, as described in the Methods. Proteins were resolved by SDS-PAGE and immunoblotted with the indicated antibodies. The result shown is representative of three independent experiments.

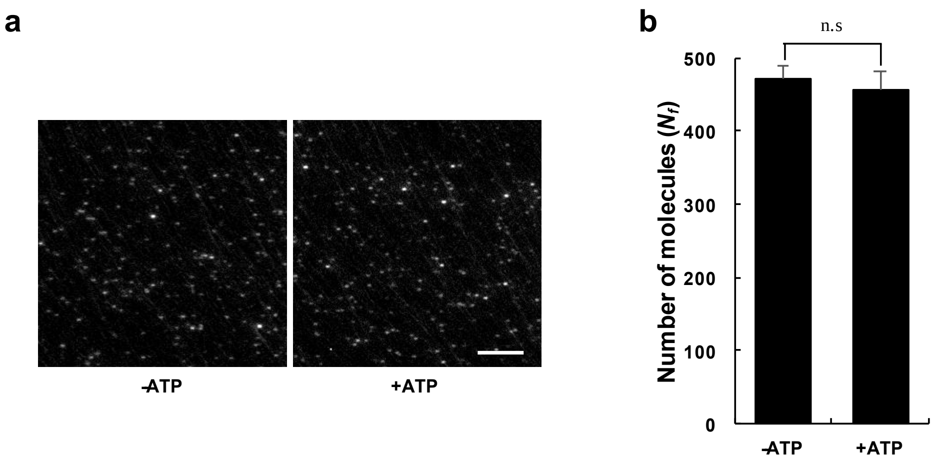


**Supplementary Figure 8. Immobilization of *in vitro* autophosphorylated EGFR**

Experiments were performed in parallel to those shown in Supplementary Figure 7. *In vitro* autophosphorylated rEGFR was applied to a single-molecule surface. (**a**) TIRF images of biotin-labeled rEGFR. Scale bar, 5 µm. (**b**) The graph shows the average numbers of fluorescent molecules per imaging area ($N_f$) with or without ATP. Error bars denote standard deviation (n > 5). n.s., not significant.

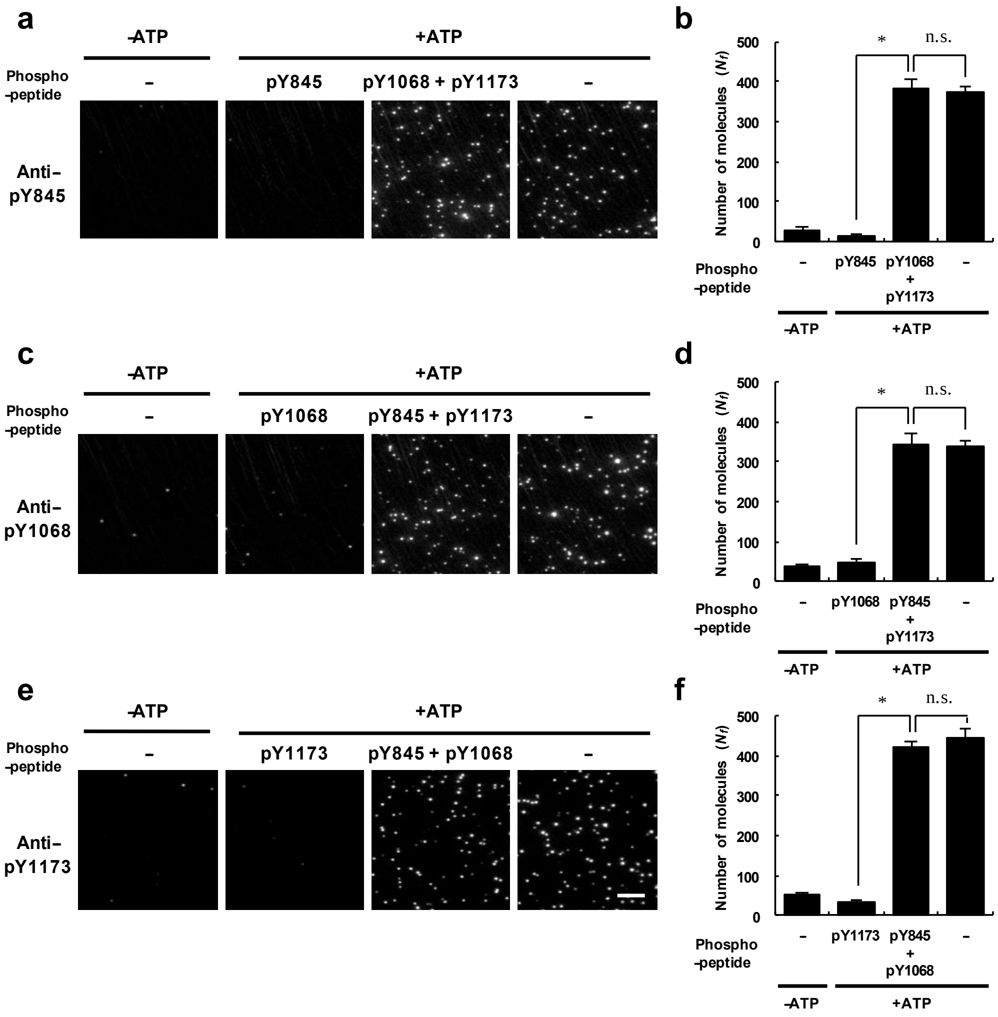


**Supplementary Figure 9. Validation of site-specific phospho-antibodies using phospho-peptides**

Experiments were performed in parallel to those shown in Supplementary Figure 7. *In vitro* autophosphorylated rEGFR was applied to a single-molecule surface. Each site-specific autophosphorylation of rEGFR was detected by the indicated phospho-antibodies (pY845, pY1068, and pY1173) with or without 0.4 μM of the indicated phospho-peptides. (**a**, **c**, **e**) Representative SiMBlot images of Cy3 signals generated by probing site-specific autophosphorylation of rEGFR with the indicated primary and fluorescently labeled secondary antibodies. Scale bar, 5 μm. (**b**, **d**, **f**) Graphs show the average numbers of fluorescent molecules per imaging area ($N_f$) using the indicated site-specific phospho-antibodies. Error bars denote standard deviation (n > 5). *, p < 0.005. n.s., not significant.

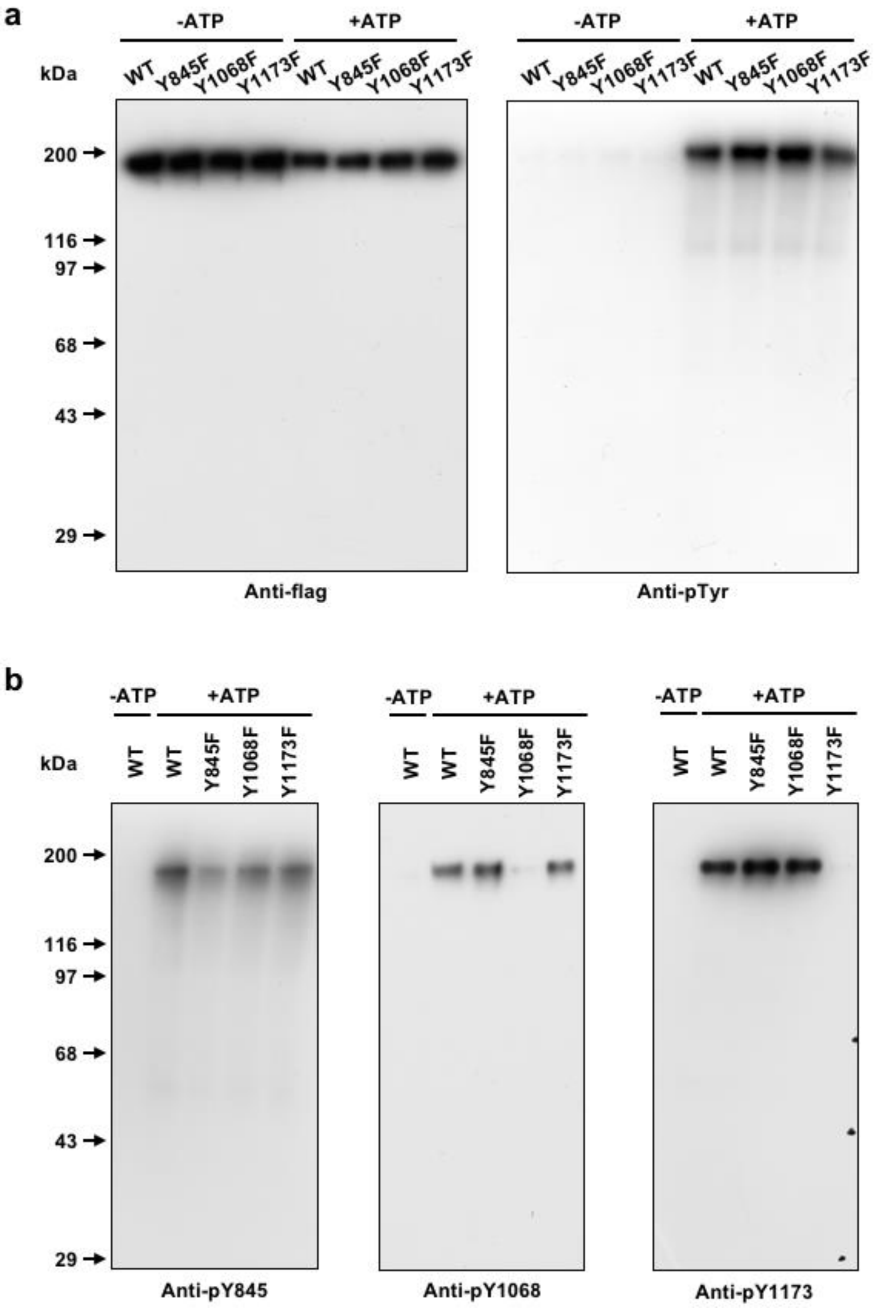
a
-ATP
+ATP
kDa
WT
Y845F
Y1068F
Y1173F
200
116
97
68
43
29
Anti-flag
Anti-pTyr
b
Anti-pY845
Anti-pY1068
Anti-pY1173

c

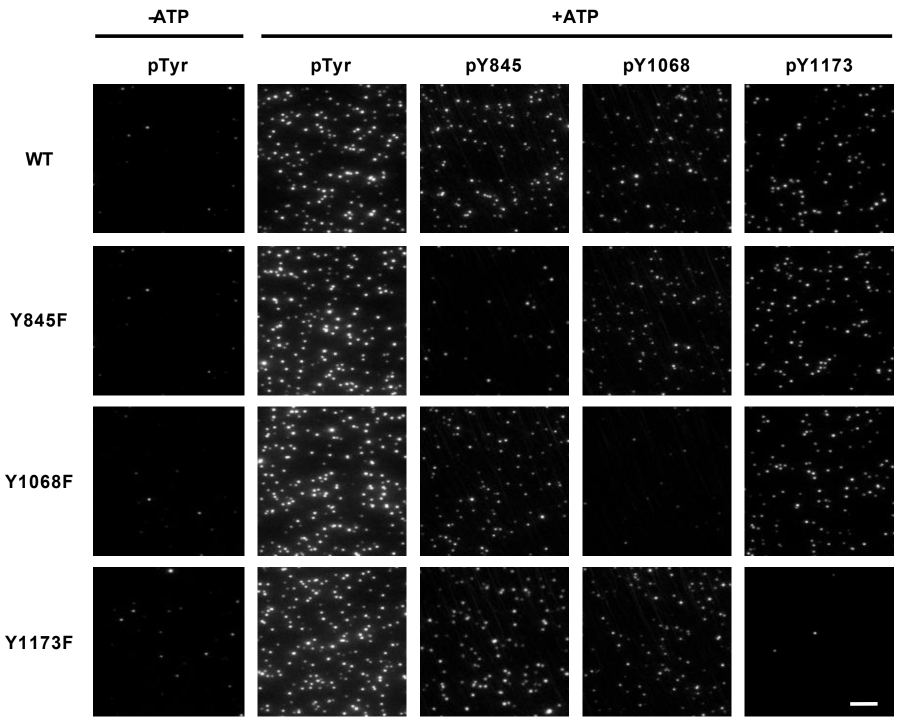


d

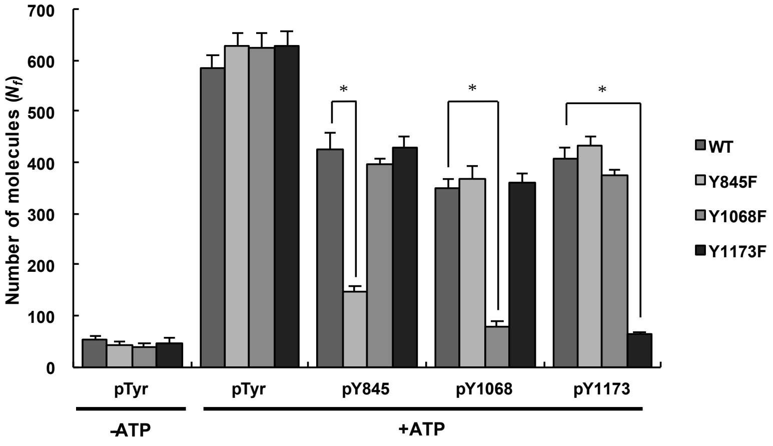


**Supplementary Figure 10. Validation of site-specific phospho-antibodies using *in vitro* autophosphorylated EGFR mutants**

After starvation for 24 h, COS7 cells expressing WT or mutant EGFR-eGFP-Flag (Y845F, Y1068F, or Y1173F) were labeled with a biotin derivative reagent and extracted with lysis buffer. For *in vitro* autophosphorylation, affinity-purified rEGFR was incubated with 100 ng $ml^{-1}$ EGF and 0.1 mM ATP, as described in the Methods. *In vitro* autophosphorylated rEGFR was subjected to immunoblotting (**a, b**) and SiMBlot (**c, d**). Each site-specific autophosphorylation of rEGFR was detected by the indicated phospho-antibodies (pTyr, pY845, pY1068, and pY1173). (**c**)

Representative SiMBlot images of Cy3 signals generated by probing site-specific phosphorylation with the indicated primary and fluorescently-labeled secondary antibodies. Scale bar, 5 μm. (**d**) Graphs show the average numbers of fluorescent molecules per imaging area ($N_f$) using the indicated site-specific phospho-antibodies. Error bars denote standard deviation ($n > 5$). *, $p < 0.05$.

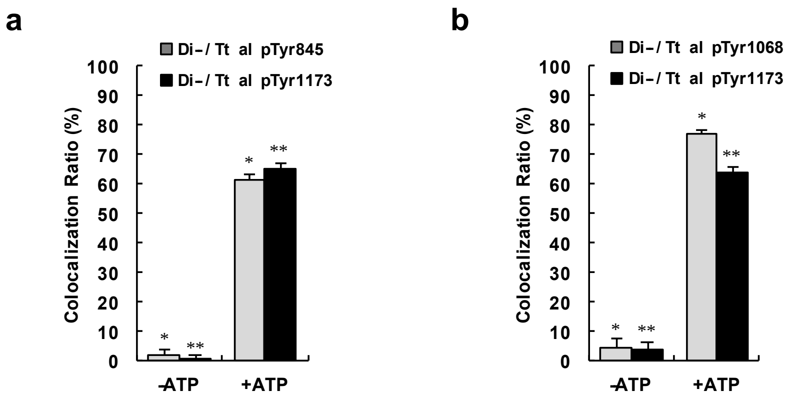


**Supplementary Figure 11. Comparative colocalization analysis of *in vitro* autophosphorylated EGFR**

Experiments were performed in parallel to those shown in Supplementary Figure 7. *In vitro* autophosphorylated rEGFR was applied to a single-molecule surface. The number of colocalized molecules divided by the total number of Alexa488 or Cy3 signals was presented as the colocalization ratio. Autophosphorylation pairs: (**a**) pTyr845/pTyr1173, (**b**) pTyr1068/pTyr1173. Error bars denote standard deviation ($n > 5$). *, **, $p < 0.005$.

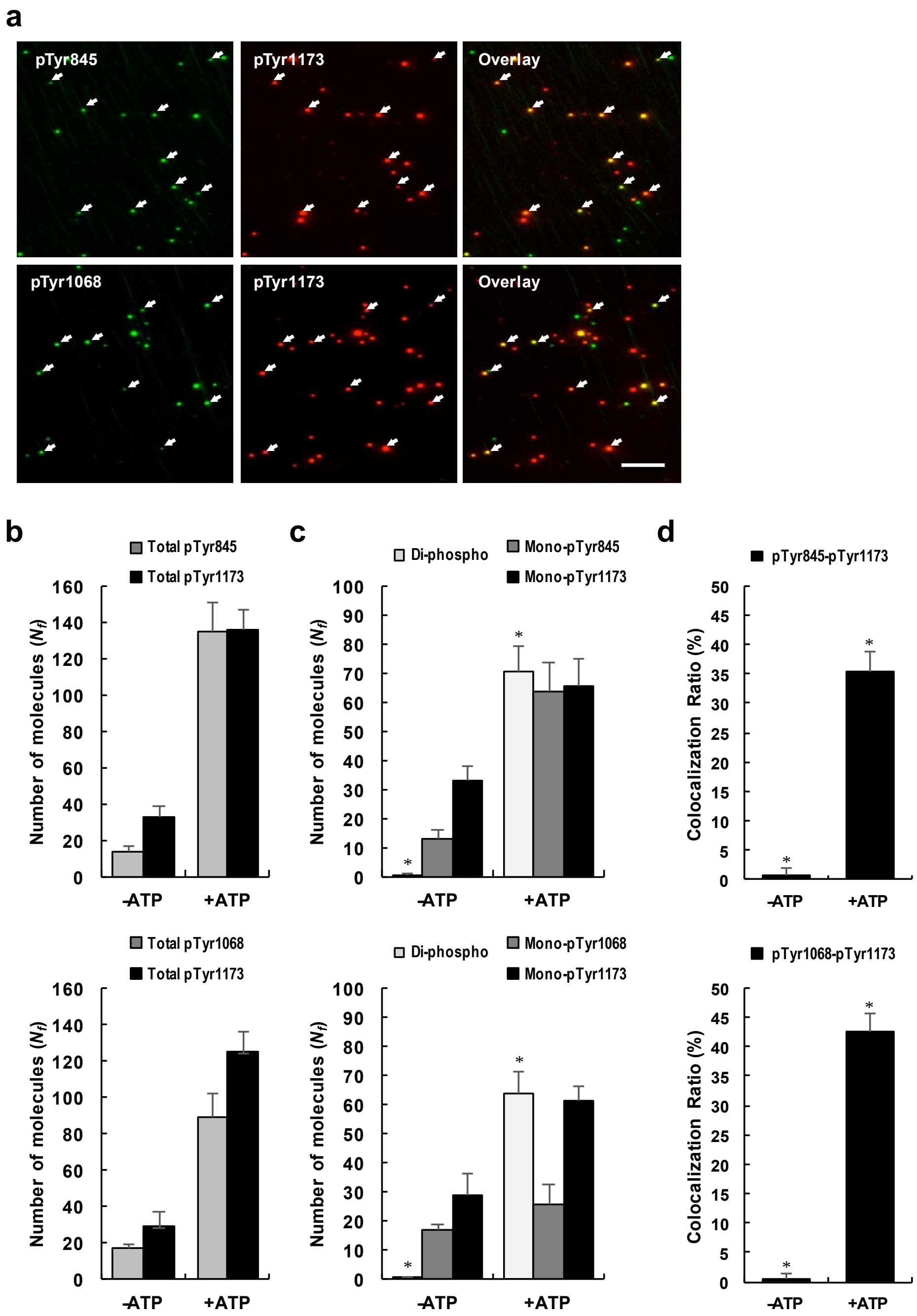


**Supplementary Figure 12. Detection of pairwise site-specific phosphorylation of *in vitro* autophosphorylated EGFR**

Experiments were performed in parallel to those shown in Figure 4. The diluted *in vitro* autophosphorylated rEGFR was applied to a single-molecule surface. Autophosphorylation sites of rEGFR were quantified by the SiMBlot assay with the indicated antibody sets. (**a**) Representative SiMBlot images of Alexa488 and Cy3 signals generated by probing site-specific phosphorylation with the indicated primary and fluorescently-labeled secondary antibodies. Scale bar, 5 μm. (**b)** Graphs show the average numbers of fluorescent molecules per imaging area ($N_f$) using the indicated

antibodies. (**c**) Pairwise phosphorylation of EGFR was analyzed by colocalization of the indicated antibody sets. (**d**) Colocalization ratio of the indicated antibody sets. Error bars denote standard deviation ($n > 5$). *, $p < 0.05$.

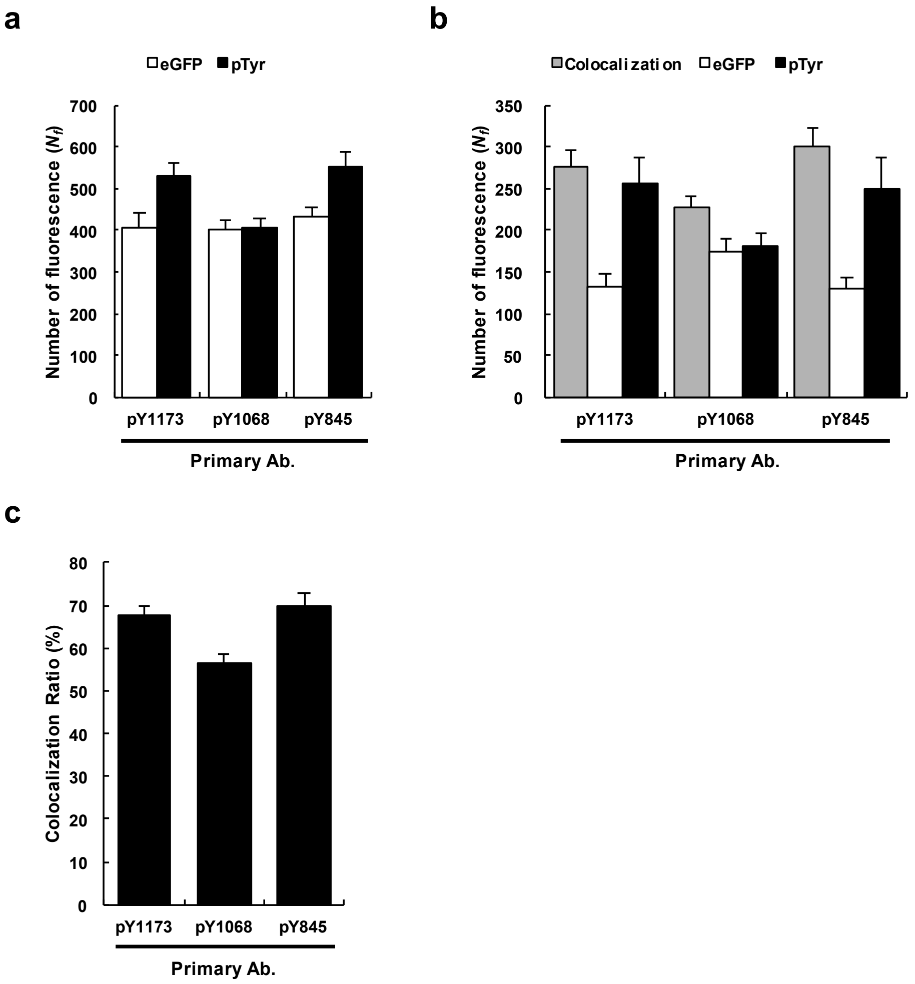


**Supplementary Figure 13. Labeling efficiency of site-specific phospho-antibodies for *in vitro* autophosphorylated EGFR**

Experiments were performed in parallel to those shown in Supplementary Figure 7. *In vitro* autophosphorylated rEGFR was applied to a single-molecule surface. eGFP signals were from immobilized rEGFR. Cy3 signals were from probing tyrosine phosphorylation using a primary antibody against site-specific tyrosine phosphorylation (pY845, pY1068, or pY1173) and a fluorescently labeled secondary antibody. (**a**) Average numbers of fluorescent molecules per imaging area ($N_f$). Error bars denote standard deviation ($n > 5$). (**b**, **c**) Colocalization numbers and ratios of each site-specific tyrosine phosphorylation on rEGFR. *, $p < 0.05$.

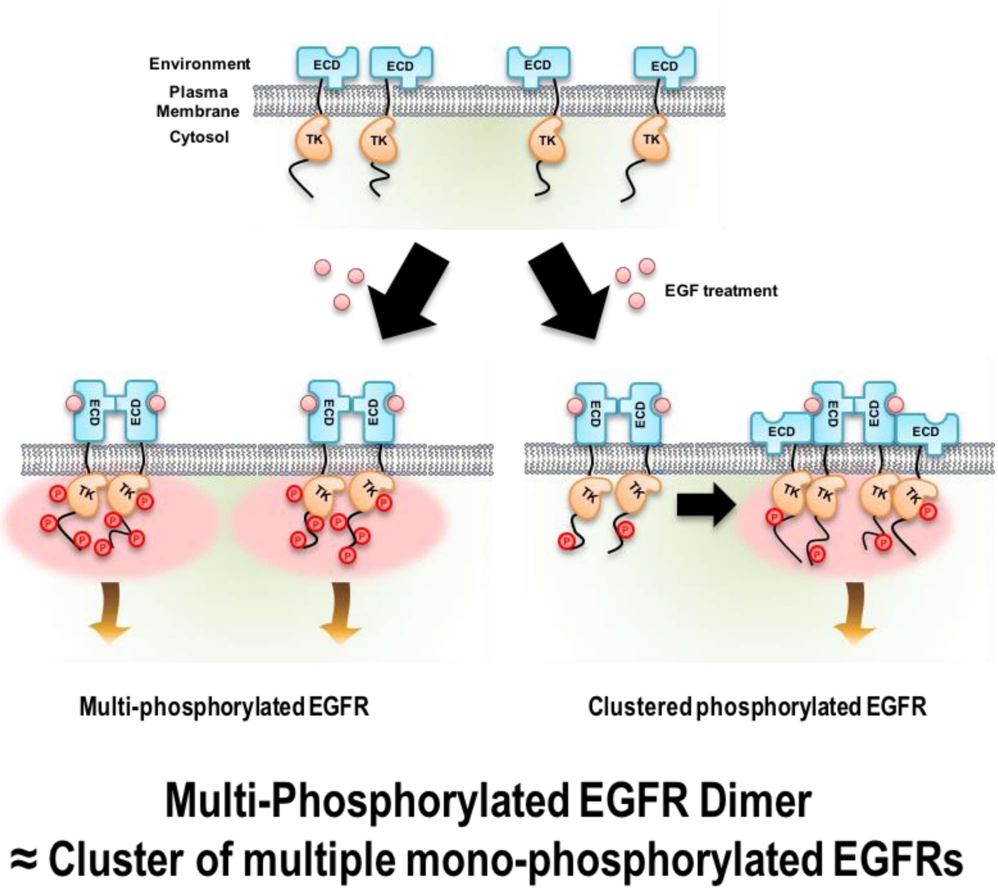


**Supplementary Figure 14. Two models of EGF-dependent EGFR signaling complex formation**

In these models, EGF induces EGFR signaling formation as the 'All-in-One' model involving a multi-functional EGFR dimer with multi-phosphorylation (left) or the 'All-for-One' model involving a set of individually functional EGFRs with mono-phosphorylation (right).

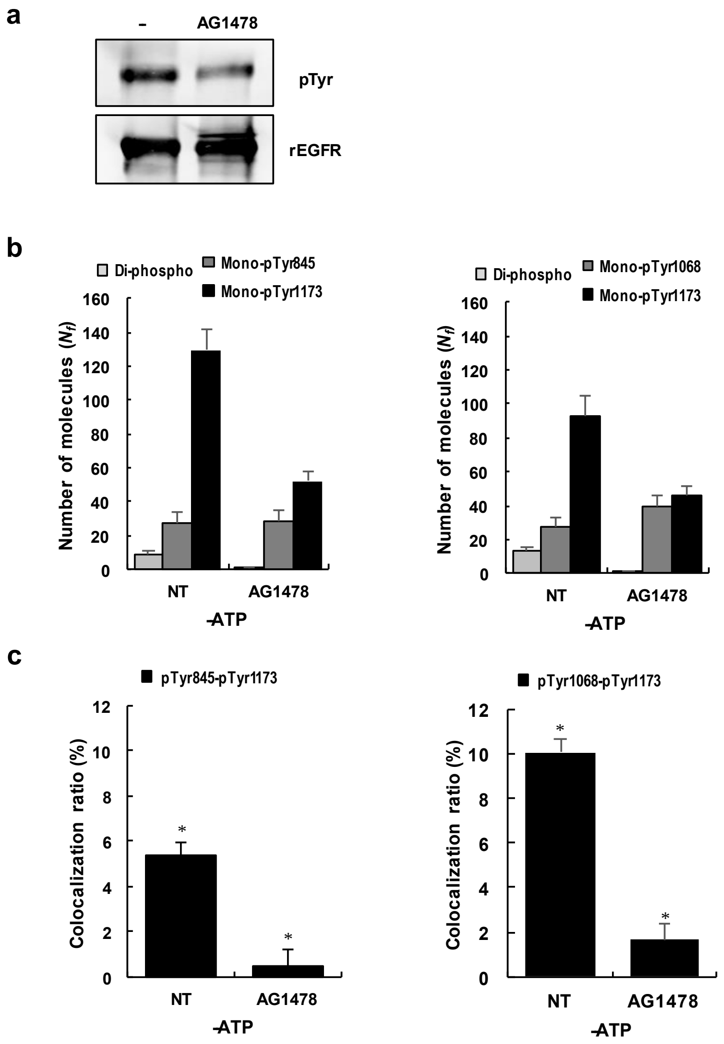


**Supplementary Figure 15. Purification of rEGFR with tyrosine kinase inhibitor**

After starvation for 24 h, COS7 cells expressing EGFR-GFP-Flag were labeled with a biotin derivative reagent and extracted with lysis buffer. rEGFR was affinity-purified using anti-FLAG M2-conjugated agarose with or without 10 μM AG1478, an EGFR kinase inhibitor. The eluted rEGFR was subjected to immunoblotting (a) and SiMBlot (b) with the indicated antibody sets. Graphs show the average numbers of fluorescent molecules per imaging area ($N_f$) using the indicated antibodies. Pairwise phosphorylation of EGFR was analyzed by colocalization of the indicated antibody sets. (c) Colocalization ratio of the indicated antibody sets. Error bars denote standard deviation (n > 5). *, $p < 0.05$.

**Supplementary Table 1. Site-specificity of phospho-antibodies on *in vitro* autophosphorylated EGFR**

| Pair | % [a] |
|---|---|
| **pTyr845** | **65.38** |
| **pTyr1068** | **77.16** |
| **pTyr1173** | **84.09** |

[a] Obtained from Supplementary Fig. 10

**Supplementary Table 2. Phosphorylation probability on *in vitro* autophosphorylated EGFR**

| Pair | Probability | % |
|---|---|---|
| **pTyr845** | **P(pTyr845)** | **45.65 ± 1.84**[a] |
| **pTyr1068** | **P(pTyr1068)** | **43.59 ± 1.76**[a] |
| **pTyr1173** | **P(pTyr1173)** | **56.79 ± 1.79**[a] |
| **pTyr845-pTyr1173** | **P(pTyr845\|pTyr1173)** | **42.68 ± 0.94**[b] |
| | **P(pTyr1173\|pTyr845)** | **51.70 ± 1.39**[b] |
| **pTyr1068-pTyr1173** | **P(pTyr1068\|pTyr1173)** | **49.05 ± 1.63**[b] |
| | **P(pTyr1173\|pTyr1068)** | **64.79 ± 1.10**[b] |

[a] Obtained from Supplementary Fig. 13 and corrected by Supplementary Table. 1

[b] Obtained from Supplementary Fig. 11 and corrected by Supplementary Table. 1

**Supplementary Table 3. Co-occurrence of pairwise phosphorylation patterns on *in vitro* autophosphorylated EGFR**

| Pair | Probability | *Lift* value[a] |
|---|---|---|
| **pTyr845-pTyr1173** | **P(pTyr845\|pTyr1173) / P(pTyr845)** | **0.93** |
| | **P(pTyr1173\|pTyr845) / P(pTyr1173)** | **0.91** |
| **pTyr1068-pTyr1173** | **P(pTyr1068\|pTyr1173) / P(pTyr1068)** | **1.13** |
| | **P(pTyr1173\|pTyr1068) / P(pTyr1173)** | **1.14** |

[a] A value ≈ 1 indicates no association between pairwise phosphorylation sites.

A value < 1 indicates that pairwise phosphorylation sites are negatively associated.

A value > 1 indicates that pairwise phosphorylation sites are positively associated.
